# Risk assessment and management of preoperative venous thromboembolism following femoral neck fracture

**DOI:** 10.1186/s13018-018-0998-4

**Published:** 2018-11-20

**Authors:** Ze-Nan Xia, Ke Xiao, Wei Zhu, Bin Feng, Bao-Zhong Zhang, Jin Lin, Wen-Wei Qian, Jin Jin, Na Gao, Gui-Xing Qiu, Xi-Sheng Weng

**Affiliations:** Department of Orthopaedics, Peking Union Medical College Hospital, Chinese Academy of Medical Sciences, Shuaifuyuan 1#, Wangfujing, Dongcheng District, Beijing, 100730 People’s Republic of China

## Abstract

**Background:**

Limited studies are available to investigate the prevalence of preoperative venous thromboembolism (VTE) in elderly patients with femoral neck fractures. Our primary aim was to determine the incidences of VTE and its risk or protective factors in such patient population. The secondary objective was to evaluate the need of therapeutic anticoagulation for isolated calf muscular venous thrombosis (ICMVT) prior to femoral neck fracture surgery.

**Methods:**

This is a retrospective case-control study, including 301 femoral neck fracture patients who were admitted to our institution between January 2014 and March 2017. Bilateral Doppler ultrasonography was performed in each of the patients as a preoperative VTE screening. The event rate of VTE was calculated, and significant risk or protective factors were determined by using a multivariate logistic regression model. Patients with ICMVT were divided into anticoagulation and no anticoagulation groups to assess the efficacy and safety of preoperative therapeutic anticoagulation. Intraoperative blood loss, drainage volume, blood transfusion, perioperative hemoglobin change, and rate of thrombosis extension were compared between the two groups.

**Results:**

The overall preoperative incidence of VTE in patients with femoral neck fracture was 18.9% (57/301), in which deep vein thrombosis (DVT) was 18.9% and pulmonary embolism (PE) was 1%. Among the DVT cases, 77.2% (44/57) were ICMVTs. Multiple fractures (odds ratio [OR] = 9.418; 95% confidence interval [CI] = 2.537 to 34.96), coexisting movement disorder (OR = 3.862; 95% CI = 1.658 to 8.993), bed rest for more than 7 days (OR = 2.082; 95% CI = 1.011 to 4.284) as well as elevated levels of D-dimer (OR = 1.019; 95% CI = 1.002 to 1.037) and fibrinogen (OR = 1.345; 95% CI = 1.008 to 1.796) led to an increase in the risk of VTE, while the recent use of antiplatelet drug (OR = 0.424; 95% CI = 0.181 to 0.995) and prophylactic anticoagulation (OR = 0.503; 95% CI = 0.263 to 0.959) decreased the risk of VTE. For the 39 patients with ICMVT undergoing femoral neck fracture surgery, there were no significant differences in the rate of thrombosis extension between anticoagulation and no anticoagulation groups, but significantly decreased postoperative hemoglobin was observed in the anticoagulation group.

**Conclusion:**

Our findings showed a high prevalence of preoperative VTE in elderly patients with femoral neck fracture, with risk factors identified. We found that the most detected VTE were ICMVTs. Our study suggested that a direct surgery without preoperative use of therapeutic anticoagulation for ICMVT would not reduce the risk of thrombus extension, and the therapeutic use of anticoagulation may worsen postoperative anemia.

## Introduction

Hip fractures have posed a significant health care problem worldwide as the elderly population is increasing. It was estimated that the number of hip fractures is approximately 1.7 million each year, and the number is expected to surpass 6 million by the year 2050 [1, 2]. Patients with hip fractures are at high risk of venous thromboembolism (VTE), including deep vein thrombosis (DVT) and pulmonary embolism (PE), which is a major cause of morbidity and mortality [3, 4]. A number of literature have focused on the risk factors and incidence of VTE developed after hip fracture surgery. The reported event rate of DVT and PE varied from 1.18 to 6% and 0.25 to 4.6%, respectively, when prophylaxis was used [5–8]. However, there was limited information on the prevalence of preoperative VTE in hip fracture patients waiting for surgery. Previous studies reported a great variation in the preoperative incidence of VTE after hip fracture ranging from 6 to 62%. These studies had several limitations including the highly selected population, small sample size, lack of thrombosis status in unaffected limbs, failure to detect calf muscular venous thrombosis by venography, and the lack of details of specific risk factors for assessment [9–15]. Identification of risk factors for VTE following hip fracture would provide important information on the prevention of thrombosis events and preoperative optimization, leading to a decreased rate of death and complications during the perioperative period. Several studies have reported that delayed admission or surgical intervention, elevated levels of D-dimer, female patients, pulmonary disease, and previous VTE were associated with an increased risk of VTE prior to hip fracture surgery [9, 11–14]. In the elderly population, femoral neck fractures occur frequently. Patients often have multiple comorbidity and concomitant medication, and we hypothesize that additional risk factors would contribute to the relatively high preoperative incidence of VTE, despite thromboprophylaxis is received.

Isolated calf muscular venous thrombosis (ICMVT) is confined to the gastrocnemius and soleal veins and accounts for 5.6 to 31.3% of DVT in lower extremities [16]. The latest American College of Chest Physicians (ACCP) guidelines recommend serial imaging of the deep veins for 2 weeks over anticoagulation in patients with isolated distal DVT without severe symptoms or risk factors for thrombosis extension and consider ICMVT is associated with a lower risk of extension than that of calf axial vein thrombosis [17]. However, the treatment protocols concerning ICMVT remain unclear, particularly in patients with hip fractures awaiting surgical treatment [18]. Preoperative therapeutic anticoagulation could effectively prevent thrombosis extension but, on the other hand, may increase the risk of perioperative bleeding and bed rest-related complications such as pneumonia, urinary infection, and decubitus ulcer. While immediate surgery without anticoagulation for ICMVT is beneficial to the earlier recovery of walking ability, the major concern remains on the possibility of proximal extension. It is important to take into consideration both the effectiveness and safety of the regimen when deciding whether therapeutic anticoagulation for ICMVT is necessary for patients awaiting elective hip fracture surgery. To date, there are no studies evaluating the outcomes of low molecular weight heparin (LMWH) treatment for ICMVT in the elderly patient population with femoral neck fracture. The purpose of the present study was to investigate the preoperative prevalence of VTE in patients with femoral neck fractures and identify associated risk or protective factors in these patients. Furthermore, this study compared the outcomes of two regimens for ICMVT and evaluated the necessity of therapeutic anticoagulation in patients waiting for femoral neck fracture surgery.

## Materials and methods

### Study population

This study was approved by the Ethics Committee of Peking Union Medical College Hospital (PUMCH), and written informed consent was obtained from all the participants prior to enrollment into the study. We conducted a retrospective review of all patients with femoral neck fracture, identified by hip joint X-ray in our institution between January 2014 and March 2017. Thirty-eight patients were excluded from the study due to the absence of evaluation information for the status of the deep venous system in lower extremities, which led to a total of 301 patients included in this study. Bilateral Doppler ultrasonography was routinely performed in each of the included patients after admission, if a DVT was identified, therapeutic anticoagulation was administrated according to the ACCP guidelines (2016, 10th edition). The insertion of an inferior vena cava filter (IVCF) before surgery was indicated when patients were at high risk of developing a life-threatening PE. Computed tomography pulmonary angiogram (CTPA) was applied to confirm a diagnosis of PE if patients presented with suggestive symptoms such as dyspnea, unexplained tachycardia, chest pain, decreased pulse oximetry, or abnormal arterial blood gas.

### Outcome measures

To determine the risk factors for the occurrence of preoperative VTE following femoral neck fracture, associated clinical-pathological characteristics were collected, including age, gender, BMI, classification of fracture, duration of bed rest, comorbidities, smoking, long-term use of steroid or immunosuppressant, recent use of antiplatelet drug (within one week before injury), prophylactic anticoagulation prior to VTE diagnosis, and preoperative level of D-dimer, fibrinogen, and inflammation markers. Investigative comorbidities included hypertension, diabetes, malignancy, ischemic heart disease, atrial fibrillation, cerebrovascular disorder, chronic kidney disease, chronic pulmonary disease, autoimmune disease, movement disorder, Alzheimer's disease, and multiple fractures. Movement disorder was defined as a status of impaired or diminished movement in lower extremities caused by various diseases involving nervous, muscular, or skeletal system, such as hemiparalysis after stroke, myasthenia gravis, Parkinson’s disease, and advanced osteoarthritis.

Patients with ICMVT undergoing fracture surgery were divided into anticoagulation and no anticoagulation groups. In the anticoagulation group, patients received therapeutic anticoagulation after detection of ICMVT from at least 12 h before surgery. In the no anticoagulation group, patients were arranged for surgery directly without anticoagulation after ICMVT diagnosis. Postoperative anticoagulation was initiated 12 h after surgery in both groups. LMWH was used as antithrombotics in each study patient at a two thirds to full dose of 100 U/kg twice daily during admission and turned to rivaroxaban after discharge for at least 3 months. Repeated Doppler ultrasonography was employed for suspected DVT if a patient developed sudden onset of lower limb swelling, pain, warmth, or erythema during 2 weeks of follow-up from the date of surgery. Baseline characteristics, efficacy, and safety of LMWH therapy were compared between the two groups. Intraoperative blood loss, drainage volume, blood transfusion, and perioperative hemoglobin change were measured to assess the safety of anticoagulation, while the rate of thrombosis extension was served as the parameter to judge whether anticoagulation was effective.

### Statistical analysis

The SPSS 19.0 software (Chicago, IL, USA) was used for statistical analysis. Categorical variables were presented as proportions and continuous variables were presented as the mean ± standard error. Chi-square test and Fisher’s exact test were used for comparisons between categorical variables, and Wilcoxon two-sample test and Student’s *t* test were used for continuous variables. Univariate analysis was performed on numerous possible risk factors associated with preoperative VTE following femoral neck fracture. Factors with significant difference (combined movement disorder, multiple fractures, fibrinogen, bed rest time, and antiplatelet therapy) were included in the multivariate logistic regression model to generate adjusted odds ratios (OR) with 95% confidence intervals (CI) for further analysis. Besides, D-dimer and prophylactic anticoagulation employed as important potential risk factors were also forced into the model. However, factors with data missing were excluded as erythrocyte sedimentation rate (ESR). Receiver operating characteristic (ROC) curve and area under the curve (AUC) analysis was used to evaluate the predictive value of risk factors. For all tests, *P* < 0.05 was considered statistically significant.

## Results

Three hundred and thirty-nine patients with femoral neck fracture were admitted to our institution between January 2014 to March 2017. After excluding 38 patients in whom Doppler ultrasonography of deep vein in lower extremities was not performed, a total of 301 patients were included in this study. Of these patients, 57 (18.9%) were identified having DVTs by Doppler ultrasonography screening before surgery, and all of them were asymptomatic. Most of the DVTs (*n* = 44, 77.2%) were ICMVTs, 5 (7%) involved true distal deep veins (peroneal, tibial), and 8 (14%) involved proximal deep vein (external iliac, common femoral, superficial femoral); 35 DVTs (61.4%) were detected on the ipsilateral side of the fracture, 15 were detected on the contralateral side, and 7 occurred on both sides. PEs were diagnosed in 3 patients with proximal DVT using CTPA. After detection of VTE, 40 patients received therapeutic anticoagulation with LMWH, and 17 underwent fracture surgery without preoperative therapeutic anticoagulation. Surgeries for fracture under general anesthesia were performed in 50 patients, including hemi-arthroplasty in 40, total hip arthroplasty (THA) in 8, and closed reduction and internal fixation with cannulated screw in 2. Temporary or permanent IVCFs were implanted in 3 patients prior to surgery.

**Fig. 1:**
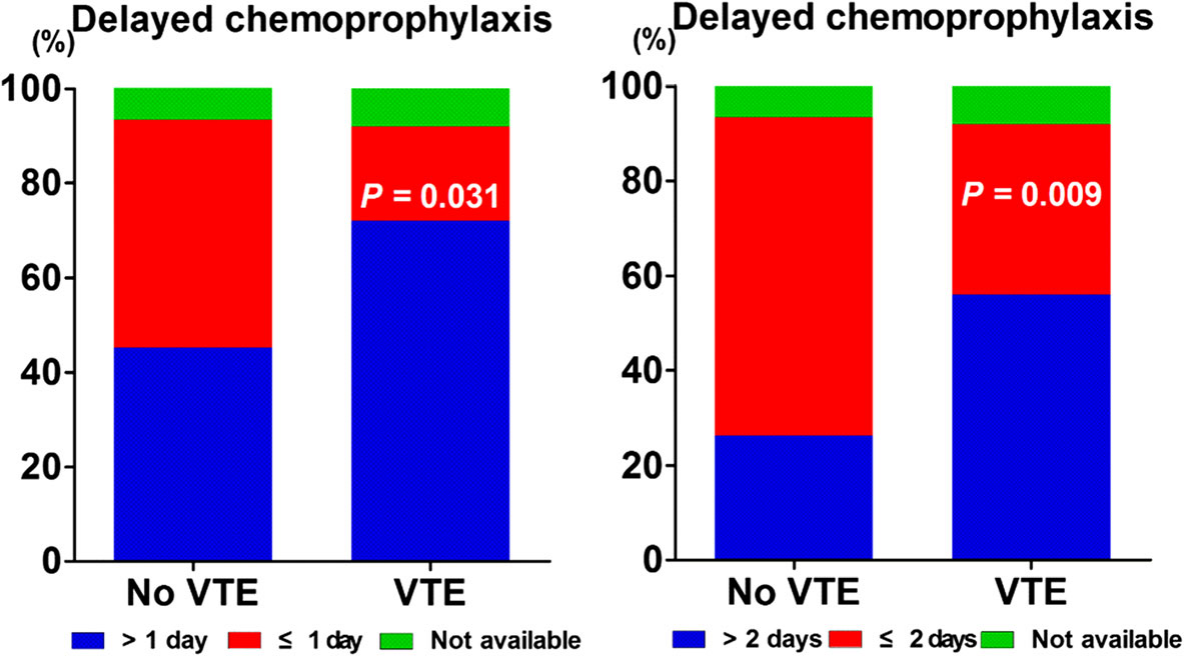
Comparison of percentage of delayed chemoprophylaxis > 1 day and 2 days between patients with and without VTE (*n* = 162). Delayed chemoprophylaxis was defined as prophylactic anticoagulation initiated at more than 24 h after femoral neck fracture

Associations of clinical-pathological parameters with preoperative VTE were listed in Table 1. In the univariate analysis, significantly elevated levels of fibrinogen and ESR were observed in patients with VTE. Although the level of D-dimer was higher in VTE group, there was no statistical difference. Duration of bed rest for more than 7 days was more commonly observed in patients with preoperative VTE, and the difference became more significant with the increase of time in bed (> 14 days). For patients with comorbidities and concomitant medication, movement disorder and multiple fractures were more frequently found in patients with VTE; in contrast, the recent use of antiplatelet drug was relatively less frequent. The proportion of prophylactic anticoagulation use tended to be higher in patients without VTE, but the difference was not significant. Notably, more patients received delayed chemoprophylaxis at more than 24 h after fracture in VTE group, and the difference became more significant when there was a delay on commencement of chemoprophylaxis for more than 48 h from the time of fracture (Fig. 1).

**Table 1:** Associations of clinical-pathological characteristics and preoperative VTE

Variable	No VTE (*n* = 244)	VTE (*n* = 57)	*P* value
Age (year)	76.30 ± 11.10	77.11 ± 9.38	0.764
Male gender	80 (32.79)	15 (26.32)	0.344
BMI (kg/m^2^)	22.91 ± 3.69	22.91 ± 4.09	0.684
Garden typing			0.071
Type III and IV	216 (88.52)	55 (96.49)	
Type I and II	28 (11.48)	2 (3.51)	
Rockwood typing			0.280
Subcapital	186 (77.82)	47 (87.04)	
Transcervical	40 (16.74)	6 (11.11)	
Basicervical	13 (5.44)	1 (1.85)	
Bed rest time > 3 days	120 (49.38)	34 (59.65)	0.163
Bed rest time > 7 days	54 (22.22)	20 (35.00)	0.043
Bed rest time > 14 days	30 (12.35)	15 (26.32)	0.008
Smoking	20 (8.20)	7 (12.28)	0.331
Comorbidity			
Hypertension	144 (59.02)	27 (47.37)	0.110
Diabetes	71 (29.10)	10 (17.54)	0.077
Malignancy	30 (12.30)	7 (12.28)	0.998
Ischemic heart disease	57 (23.36)	8 (14.04)	0.123
Atrial fibrillation	19 (7.79)	5 (8.77)	1.000
Cerebrovascular disorder	44 (18.03)	6 (10.53)	0.170
Chronic kidney disease	12 (4.92)	3 (5.26)	1.000
Chronic pulmonary disease	10 (4.10)	3 (5.26)	0.978
Autoimmune disease	11 (4.51)	3 (5.26)	1.000
Movement disorder	18 (7.38)	14 (24.56)	< 0.001
Alzheimer's disease	7 (2.87)	4 (7.02)	0.267
Multiple fractures	6 (2.46)	7 (12.28)	0.003
Concomitant medication			
Steroid or immunosuppressant	14 (5.74)	2 (3.51)	0.728
Antiplatelet drug	66 (27.05)	8 (14.04)	0.040
Prophylactic anticoagulation	137 (56.85)	25 (43.86)	0.077
D-dimer (μg/mL)	10.11 ± 17.27	11.35 ± 22.88	0.067
Fbg (g/L)	3.92 ± 1.11	4.2 ± 1.28	0.035
ESR (mm/h)	37.35 ± 26.85	45.33 ± 25.97	0.035
hsCRP (mg/L)	42.84 ± 36.21	54.63 ± 48.69	0.142

*Fbg* fibrinogen, *ESR* erythrocyte sedimentation rate, *hsCRP* high-sensitive C-reactive protein

Seven selected variables were determined to be an independent risk or protective factors associated with preoperative VTE following femoral neck fracture in the multivariate logistic regression analysis (Table 2). Elevated levels of D-dimer and fibrinogen were significantly associated with an increased risk of VTE. Bed rest for more than 7 days led to a twofold increase in the risk of VTE, and patients with comorbidity of movement disorder or multiple fractures were at four- to ninefold higher risk for VTE. Conversely, the recent use of antiplatelet drug and prophylactic anticoagulation were found to be protective factors which significantly decreased the risk of VTE after femoral neck fracture.

**Table 2:** Multivariate logistic regression analysis for VTE

Variable	OR	95% *CI*		*P* value
		Lower limit	Upper limit	
D-dimer	1.019	1.002	1.037	0.0323
Fbg	1.345	1.008	1.796	0.0442
Multiple fractures (yes vs no)	9.418	2.537	34.960	0.0008
Movement disorder (yes vs no)	3.862	1.658	8.993	0.0017
Use of antiplatelet drug (yes vs no)	0.424	0.181	0.995	0.0487
Prophylactic anticoagulation (yes vs no)	0.503	0.263	0.959	0.0370
Bed rest time > 7 days (yes vs no)	2.082	1.011	4.284	0.0465

**Fig. 2:**
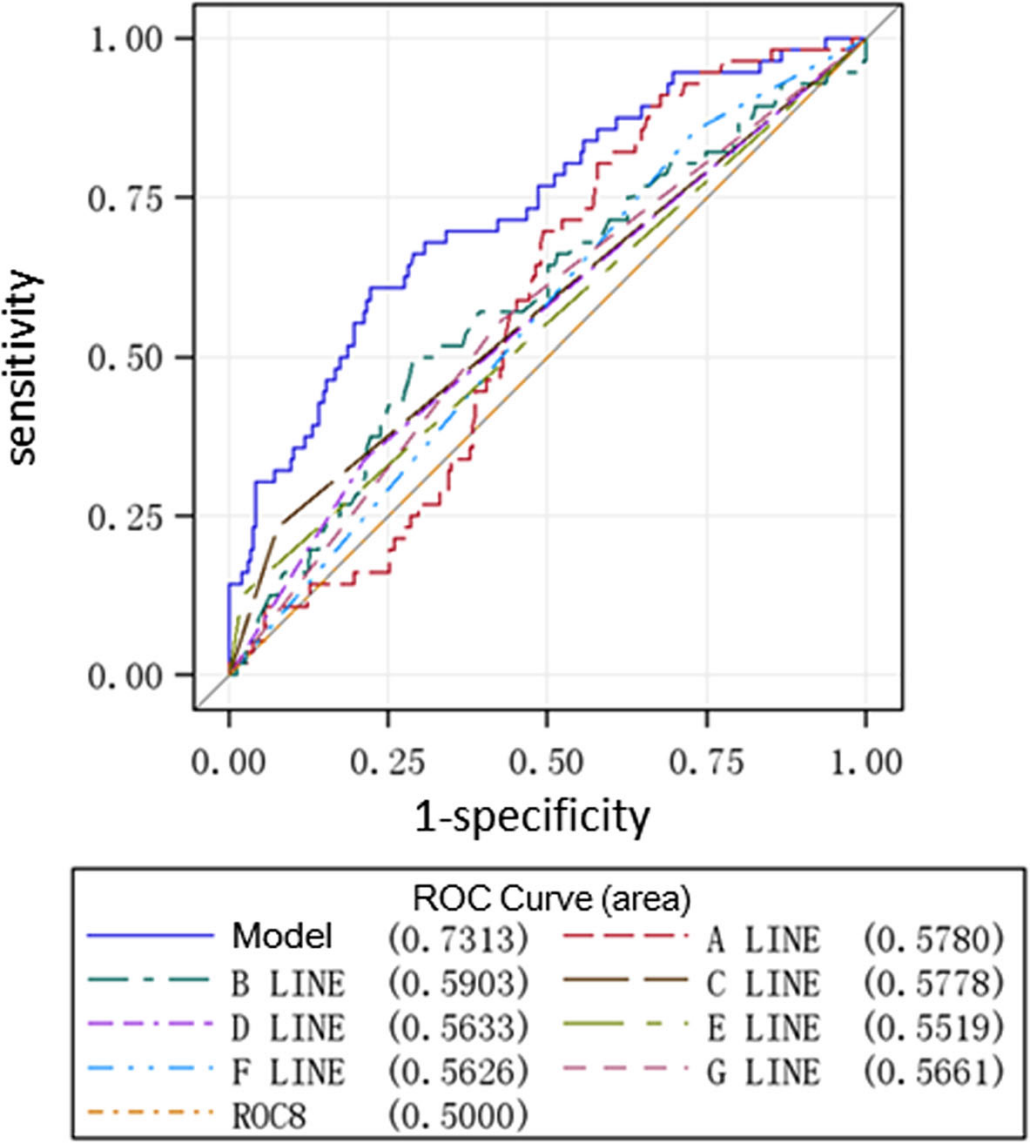
ROC curve analysis and AUC calculation for seven factors of VTE. A: D-dimer, B: fibrinogen, C: movement disorder, D: bed rest time > 7 days, E: multiple fractures, F: use of antiplatelet drug, G: prophylactic anticoagulation

ROC curve analysis was used to evaluate the potency of the identified seven risk or protective factors in predicting the occurrence of VTE following femoral neck fracture (Fig. 2). The AUC of D-dimer was 0.578 (95% IC 0.506–0.650), fibrinogen was 0.590 (95% IC 0.505–0.676), multiple fractures was 0.552 (95% IC 0.507–0.597), movement disorder was 0.578 (95% IC 0.519–0.636), antiplatelet drug use was 0.563 (95% IC 0.508–0.617), prophylactic anticoagulation was 0.566 (95% IC 0.493–0.639), and bed rest time for over 7 days was 0.563 (95% IC 0.495–0.631). Furthermore, the AUC of the established model containing all the seven factors was 0.731 (95% IC 0.657–0.806), indicating a moderate predictive value for the onset of preoperative VTE after femoral neck fracture.

Among the subpopulation of 44 patients with ICMVT, surgeries for femoral neck fracture were performed in 39 of them. Of these, 24 patients received preoperative therapeutic anticoagulation with LMWH after detection of ICMVT, and 15 underwent surgeries directly without preoperative anticoagulation. No significant differences were found in the baseline characteristics between the anticoagulation group and no anticoagulation group (Table 3). During the period of the 2-week follow-up, three ICMVTs extended into axial DVTs in the anticoagulation group; however, the difference in the rate of thrombosis extension was not statistically significant between the two groups. With respect to hemorrhage related safety assessment, intraoperative blood loss, postoperative drainage volume, and blood transfusion rate in the anticoagulation group were equivalent with those in the no anticoagulation group, but significantly decreased hemoglobin was observed after surgery in the presence of preoperative therapeutic anticoagulation. This result indicated that therapeutic anticoagulation for ICMVT prior to femoral neck fracture surgery seemed to not provide a significant reduction in the rate of thrombus extension, but in turn had a significant influence on postoperative hemoglobin.

**Table 3:** Baseline characteristics of patients with ICMVT in anticoagulation and no anticoagulation groups

Variable	Anticoagulation (*n* = 24)	No anticoagulation (*n* = 15)	*P* value
Age (year)	76.17 ± 8.99	79.33 ± 10.66	0.188
Male gender	5 (20.83)	4 (26.67)	0.711
Garden typing			0.266
Type IV	20 (83.33)	10 (66.67)	
Type III	4 (16.67)	5 (33.33)	
Pre. HGB (g/L)	120.50 ± 16.82	119.80 ± 18.59	0.904
Prophylactic anticoagulation	9 (37.50)	8 (53.33)	0.508
Surgery			0.121
Hemiarthroplasty	17 (70.83)	14 (93.33)	
Others	7 (29.17)	1 (6.67)	

*Pre.* preoperative

## Discussion

In this retrospective case-control study, we have made some major findings. Firstly, we observed the incidence of VTE following femoral neck fracture was 18.9%, independent of surgical treatment. Secondly, we identified fibrinogen, combined movement disorder, multiple fractures as new risk factors, and oral antiplatelet therapy and prophylactic anticoagulation as protective factors for the development of VTE after fractures. Finally, it is the first time that we evaluated the necessity of therapeutic anticoagulation for ICMVT in patients awaiting for femoral neck fracture surgery in terms of effectiveness and safety and revealed preoperative anticoagulation might not significantly prevent postoperative thrombus progression, but increased the risk of hemorrhage to some extent.

Patients with hip fractures have a high risk for VTE events because of endothelial injury of adjacent blood vessels, hypercoagulability following coagulation cascade, and venous stasis resulting from immobilization [19]. To date, the understanding of the preoperative VTE rate occurred after hip fracture is limited. There are only several small studies investigated that, and the findings were not consistent [9–15]. The studies employed venography for diagnosis, and the overall rate of preoperative VTE was 9.8–11.1%, or it could be as high as 29.6% when the enrolled population was limited to patients identified as subcapital femoral neck fracture without chemical thromboprophylaxis with LMWH [9, 13, 14]. Other studies showed an 11.9–16.3% overall incidence of VTE event, when Doppler ultrasonography was used [12, 15]. With the developments in venous duplex technology, the ability to detect ICMVT has improved significantly. Doppler ultrasonography has been demonstrated to be more sensitive and specific in detection of both proximal and distal DVT compared to venography, and given this, the event rate obtained by venography in earlier studies might be underestimated [20]. In addition, the highly selected population was likely to magnify or minify the real rate of thrombosis. Precluding these limitations, this study revealed the overall preoperative incidence of VTE after femoral neck fracture was 18.9% based on a larger sample size. In this respect, our result was considered to be more reliable.

Regarding the distribution of thrombosis, most were confined to calf intermuscular veins, but the proportion of 77.2% observed in this study was much higher than previous data. The variation could be mainly attributed to the different study population. We focused on the patients after femoral neck fracture who received ultrasonography screening, while population enrolled in earlier studies were patients presenting with symptoms and signs suspicious for DVT which undoubtedly led to a lower proportion of ICMVT [16]. Considering the high incidence of VTE and overwhelming percentage of asymptomatic ICMVT after femoral neck fracture, we proposed that routine screening for DVT in lower extremities with Doppler ultrasonography prior to fracture surgery be required. Notably, DVTs in our study were discovered mostly on the ipsilateral side of the fracture, and a similar finding has shown a significant tendency for DVTs to occur on the same side as the hip fracture, suggesting local vessel injury and immobility of affected limb may be more important than general hypercoagulability in causing DVT after hip fracture [21].

Although previous studies indicated the viability of incidence rate of VTE, there seemed to be a correlation between the period of delay in treatment and the incidence of perioperative thrombosis. It was observed that the rate of VTE event increased from 6 to 14.5% if patients were admitted or received surgery within 48 h to 17–62% when there was a delay for more than 48 h [9–12]. Delayed admission to hospital or surgical intervention was considered to increase the risk of preoperative VTE after hip fracture [9, 12]. Delay may result from preoperative medical evaluation and optimization as well as prolonged bed rest at home. Venous hypostasis associated with bed rest or prolonged immobilization was an important risk factor for the development of thrombosis in patients with leg injury, particularly hip fractures [13, 22], but no studies revealed the relation between the period of bed rest and risk of VTE events. We found bed rest for more than 7 days, as an independent risk, led to a twofold increase in the risk of preoperative VTE. Combined with previous findings, we believed that surgery should be performed as soon as possible, no longer than 7 days after femoral neck fracture if necessary medical optimization is required.

The present study has investigated a number of baseline characteristics and its relation to preoperative VET. We identified the elevated levels of D-dimer as one of the independent risk factors for VTE. This was consistent with another research in which the sensitivity and specificity of D-dimer in diagnosing preoperative DVT were 71.4 and 78.6% when the cutoff value was set at 2.79 mg/L [13]. D-dimer represented an applicable modality with a high sensitivity and poor specificity to detect acute DVT in nontraumatic settings. Since tissue injury caused by trauma also led to elevated levels of D-dimer, the diagnostic reliability was not validated in patients after trauma [23]. In recent years, Yang et al. have reported a cutoff value of 3 mg/L for D-dimer to predict DVT event with a sensitivity of 88.37% and a specificity of 96.96% in patients after surgery for lower limb fractures [24], and Bakhshi et al. have found that D-dimer levels more than 1000 ng/mL were 100% sensitive and 71% specific for detecting DVT in the same population [25]. All these findings suggested a yet unexplored value for D-dimer to predict VTE in patients after trauma such as femoral neck fractures.

In addition to D-dimer, high level of fibrinogen was also recognized as one of the well-established risk factors for VTE [26, 27], and it was supported by our findings in patients with femoral neck fractures for the first time. It is possible that the elevated fibrinogen increases fibrin network density, blood viscosity, and the resistance of clots to fibrinolysis, thereby leading to a hypercoagulable state [27–29]. We hypothesized that hyperfibrinogenemia is not merely a biomarker of thrombotic risk, but also contributes to the etiology of VTE following femoral neck fracture [30].

To the best of our knowledge, the relationship between ESR and VTE has never been established in patients with hip fractures. In this study, a significantly elevated level of ESR was observed in patients with VTE. This could be regarded as a potential risk factor and warrants further investigation. Fibrinogen is referred as an acute phase reactant (APR) that accompanies inflammation and tissue injury, ESR is an indirect measure of the acute phase response and levels of APR, particularly with fibrinogen, and they both reflect the presence and intensity of an inflammatory process. The increase of both ESR and fibrinogen observed in the present study implicated that the enhanced acute phase response accompanied with acute inflammatory states may play a role in the occurrence of VTE after femoral neck fracture.

Different from the previous studies, we found the development of VTE after hip fracture was not influenced by patient’s gender, type of fractures, or pulmonary disease [14], but it was significantly correlated with the coexisting movement disorder or multiple fractures. Patients with movement disorder or multiple fractures were at four- to ninefold higher risk for VTE, and it means they may need a more aggressive initial treatment and close surveillance.

LMWH or aspirin was recommended to use in patients undergoing hip fracture surgery according to the latest ACCP guidelines [18]. Sufficient evidences confirmed that chemical thromboprophylaxis resulted in a lower risk of VTE after hip fracture surgery [18, 31]. Consistently, we determined the recent use of antiplatelet drug and prophylactic anticoagulation to be protective factors which significantly reduced the risk of preoperative VTE after femoral neck fracture. For the timing of commencement of VTE prophylaxis, the guidelines recommended the initiation of LMWH therapy either from at least 12 h before surgery, or at least 12 h after surgery, but no precise protocol was given [18]. On the ground of proofs that hypercoagulable state occurred in the early phase of injury and the risk of VTE increased with the delayed period of surgical intervention, it is reasonable to initiate the thromboprophylaxis as early as possible [12, 32]. Delayed initiation of prophylaxis has been well demonstrated to contribute to the development of symptomatic VTE and fatal PE in patients with traumatic brain injury [33, 34]. A similar phenomenon was noted in this study that delayed chemoprophylaxis at more than 24 h from the time of injury was positively correlated with the occurrence of VTE, and more significant correlation was present when the delayed time was over 48 h. Thus, we suggested the use of LMWH or antiplatelet drug for VTE prophylaxis, when hip fracture surgery was delayed. The initial prophylactic anticoagulation with LMWH within 24 h appeared beneficial, while further studies are needed to determine the optimal timing of prophylactic anticoagulation commencement.

ICMVT was considered to have a lower risk of extension than thrombosis that involved the axial veins, as suggested by the ACCP guidelines [17]. However, the treatment options for ICMVT remains controversial in regard to the use of controversies monitoring with duplex examinations versus therapeutic anticoagulation. Despite the multiple trials that showed the potential benefit of anticoagulation use in the treatment of ICMVT [16, 35], a latest meta-analysis demonstrated insignificant outcomes of anticoagulation use in decreasing the rate of thrombosis progression, as well as increasing the rate of complete recanalization when compared with the no anticoagulation [36] (Table 4). The present study provided novel findings on the assessment of efficacy and safety of therapeutic anticoagulation for ICMVT in patients after femoral neck fracture. We found preoperative therapeutic anticoagulation for ICMVT may not effectively decrease the risk of thrombus progression, but may worsen postoperative anemia. As such, direct surgery for femoral neck fracture regardless of the status of ICMVT seems appropriate. We speculated that these patients could not benefit from the preoperative therapeutic anticoagulation, possibly due to the prolonged duration of bed rest, delayed recovery of walking ability, and extended inpatient time accompanied by treatment of ICMVT.

**Table 4:** Comparison of outcomes between anticoagulation and no anticoagulation groups in patients with ICMVT

Variable	Anticoagulation (*n* = 24)	No anticoagulation (*n* = 15)	*P* value
Intra. blood loss (mL)	277.08 ± 173.19	230.00 ± 154.46	0.287
Total drainage volume	204.00 ± 114.01	226.25 ± 156.75	0.911
Intra./post. blood transfusion	9 (37.50)	4 (26.67)	0.728
Post. lowest HGB	88.88 ± 13.91	98.87 ± 13.84	0.035
Peri. HGB change	31.63 ± 14.75	20.93 ± 16.92	0.044
Post. VTE extension	3 (12.50)	0 (0.00)	0.271
Post. infection	6 (25.00)	2 (13.33)	0.450

*Intra.* intraoperative, *Post.* postoperative, *Pre.* preoperative

This study had several limitations. Firstly, it was a retrospective study that relied on data from the electronic medical record system. Inferior accuracy and integrity of collected clinical information was inherent to retrospective nature compared with the prospective study. Because some data were missing such as BMI, ESR, high sensitive C-reactive protein (hsCRP), we chose to use available case analysis and to disregard some data. History of previous VTE, traction therapy, and plaster immobilization were not analyzed as associated variables due to information default. The incomplete information may affect our result to some extents. Secondly, because the data were from a single institution, selection biases was inevitable; however, the interpretation of the results would not be complicated by the heterogeneity of practices that are commonly found in a multicenter study. Thirdly, the target sample size of ICMVT was small. Lastly, we followed up thrombus progression for only 2 weeks after surgery and then performed ultrasonography only in patients with suspicious symptoms. Any long-term and asymptomatic thrombus progression events might be missed. A further study with a larger population on the evaluation of therapeutic anticoagulation for ICMVT before hip fracture surgery is greatly needed.

## Conclusion

In conclusion, we showed that the overall preoperative incidence of VTE following femoral neck fracture was high, and most of which were ICMVTs. The elevated levels of D-dimer and fibrinogen, coexisting movement disorder, and multiple fractures, as well as bed rest for more than 7 days, were identified as risk factors of VTE, while the recent use of antiplatelet drug and prophylactic anticoagulation were considered as protective factors. When ICMVT is observed after femoral neck fracture, direct surgery without therapeutic anticoagulation seems appropriate. The absence of preoperative use of therapeutic anticoagulation would not reduce the risk of thrombus extension, but it may provide less effect on postoperative anemia. This study proposed intervention strategies to lower the VTE risk and, of importance, opens a new prospect to the time of prophylactic anticoagulation commencement and management option of ICMVT.

## Abbreviations

ACCP: American College of Chest Physicians
AUC: Area under the curve
CI: Confidence intervals
CTPA: Computed tomography pulmonary angiogram
DVT: Deep vein thrombosis
ESR: Erythrocyte sedimentation rate
hsCRP: High sensitive C-reactive protein
ICMVT: Isolated calf muscular venous thrombosis
IVCF: Inferior vena cava filter
LMWH: Low molecular weight heparin
OR: Odds ratios
PE: Pulmonary embolism
ROC: Receiver-operating characteristic
THA: Total hip arthroplasty
VTE: Venous thromboembolism
